# A new discovery of STAT4 single nucleotide polymorphisms associated with hepatocellular carcinoma risk in Chinese Han population: a case–control study

**DOI:** 10.1042/BSR20210124

**Published:** 2021-07-26

**Authors:** Xu Chao, Jieqiong Wu, Wei Zhang, Xuesong Feng, Luyan Zhao, Feng Huang, Chao Jiang

**Affiliations:** 1 The Second Affiliated Hospital, Shaanxi University of Chinese Medicine, Xianyang 712000, Shaanxi Province, China; 2 The College of Basic Medicine, Shaanxi University of Chinese Medicine, Xianyang 712000, Shaanxi Province, China; 3 Pharmaceutical Factory, Shaanxi University of Chinese Medicine, Xianyang 712000, Shaanxi Province, China; 4 The Third Department of Neurology, The Second Affiliated Hospital of Xi’an Medical University, Xi’an710038, Shaanxi Province, China

**Keywords:** case-control study, hepatocellular carcinoma, single nucleotide polymorphisms, STAT4

## Abstract

**Background:** Hepatocellular carcinoma (HCC) is a common fatal malignant tumor worldwide. Signal transducer and activator of transcription 4 (STAT4) is HCC susceptibility gene identified by genome-wide association study. The purpose of the present study was to determine the association between four candidate single nucleotide polymorphisms (SNPs) in STAT4 genes and HCC risk in Chinese Han population.

**Methods:** A case–control study was conducted to assess the association between STAT4 SNPs and HCC risk in 1011 Chinese Han population. Agena MassARRAY was used to genotype SNPs. The association between SNPs and HCC susceptibility under different genetic models was evaluated by logistic regression analysis. Multifactorial dimension reduction (MDR) analyzed the interaction of ‘SNP–SNP’ in HCC risk. The difference of clinical characteristics between different genotypes was completed by ANOVA.

**Results:** The results showed that STAT4 rs11889341 was significantly associated with HCC risk under multiple genetic models (homozygote: odds ratio (OR) = 0.60, *P*=0.033; recessive: OR = 0.63, *P*=0.028; log-additive: OR = 0.83, *P*=0.032). The results of subgroup analysis showed that STAT4 rs11889341 is significantly associated with HCC risk with participants who were >55 years, male or smoking. Both STAT4 rs7574865 and rs10174238 were significantly associated with HCC risk among participants who were >55 years, smoking or drinking. STAT4 haplotype (T_rs11889341_T_rs7574865_) could reduce the risk of HCC. In addition, rs11889341 and rs7574865 were significantly associated with the level of serum ferritin (SF).

**Conclusion:** STAT4 rs11889341, rs7574865 or rs10174238 is potentially associated with HCC risk in Chinese Han population. In particular, rs11889341 showed outstanding association with HCC risk.

## Introduction

Primary hepatocellular carcinoma (abbreviated as ‘HCC’) is a common fatal malignant tumor worldwide, and more than half of the patients have been diagnosed in the middle and advanced stages [1]. HBV infection is the main cause of HCC. Past studies have shown that more than 80% of HCC patients have persistent HBV infection [2–4]. The occurrence and development of cancer is a complex process with multiple factors, multiple genes and multiple stages. It is the result of the combined effect of genetic factors and environmental factors. In recent years, with the development and progress of molecular biology and molecular epidemiology, a number of studies have successively identified genetic variants associated with the occurrence and development of HCC through genome-wide association studies. These genetic variants can not only assess the HCC susceptibility, but also affect the development of HCC [5–7].

Signal transducer and activator of transcription 4 (STAT4) encoding protein is a key transcription factor of JAK/STAT signaling pathway [8]. STAT4 can be activated by IL-12, regulating the release of inflammatory mediators by T cells and NK cells. STAT4 plays an important biological function in the process of cell differentiation, proliferation, invasion and metastasis induced by cytokines, which participates in the occurrence and development of various diseases such as inflammation and tumors [9]. Studies have shown that abnormal expression of STAT4 may affect the occurrence of lung cancer, gastric adenocarcinoma, liver cancer and other tumors [10,11]. STAT4 was identified as a genetic susceptibility gene for HCC by genome-wide association analysis [6]. Therefore, the research on the association between STAT4 genetic polymorphism and the susceptibility of HCC has attracted more and more attention. Many studies have reported that STAT4 gene polymorphisms have a certain association with the susceptibility of HCC, especially the association between STAT4 rs7574865 and the susceptibility of HCC has been reported many times [12–15]. However, the results of their research are not exactly the same, so the molecular mechanism of HCC susceptibility has not yet been clarified. Therefore, it is necessary to expand the scope of research and conduct studies on the association between STAT4 gene polymorphism and HCC susceptibility among different populations. This will help to lay a good foundation for the studies on the molecular mechanism of STAT4 in HCC susceptibility.

Therefore, the present study took the Chinese Han population as the study subject, and four candidate STAT4 single nucleotide polymorphisms (SNPs; rs3821236 A/G, rs11889341 T/C, rs7574865 T/G, rs10174238 G/A) were selected. The association between the candidate SNPs and the HCC susceptibility in the Chinese Han population will be evaluated. Our study will provide data supplements for the study of the association between STAT4 gene polymorphism and HCC susceptibility in the Chinese Han population. It will also provide new evidence for predicting the targeted therapy and exact molecular mechanism of STAT4 in HCC.

## Materials and methods

### Study subjects

The present study recruited 505 HBV-HCC patients as the case group (HCC group) from the outpatient or inpatient department of the Second Affiliated Hospital of Shaanxi University of Chinese Medicine. The patients were diagnosed with HBV-HCC through histopathological confirmation, liver puncture, clinical manifestations, imaging and other examinations. During the same period, we recruited 506 healthy individuals from the health examination center of the same hospital as the control group. The inclusion criteria of healthy individuals are as follows: no history of other complicated diseases; basic information about age, gender and ethnicity was not significantly different from the case group (excluding the differences in the distribution of exposure factors between case and control group caused by confounding factors). The present study adopted a ‘case–control’ experimental design as a whole. A study on the association between 4 STAT4 candidate SNPs and the HCC risk in the Chinese Han population was conducted among 1011 participants. We conducted a questionnaire survey of all participants through professional doctors. The survey content included demographic and epidemiological information (age, gender, smoking/drinking status etc.). Finally, after obtaining the informed consent of all participants, we collected their peripheral blood samples for DNA extraction. Our research was approved by ethics committee of the Second Affiliated Hospital of Shaanxi University of Chinese Medicine.

### Selection of SNPs

After consulting the relevant literature and the STAT4 gene polymorphism data in the SNP database, the SNPs with the minor allele frequency ≥ 5% in the research population were selected. Finally, four STAT4 gene polymorphisms (rs3821236 A/G, rs11889341 T/C, rs7574865 T/G, rs10174238 G/A) were selected as candidate SNPs for the present study.

### DNA extraction and genotyping

We performed DNA extraction and purification from peripheral blood samples according to the instructions of the kit (GoldMag Co. Ltd. Xi’an, China). The purified DNA was stored in the refrigerator. All primers in the present study were designed by MassARRAY Assay Design software. We used the MassARRAY system (Agena, San Diego, CA, U.S.A.) to genotype SNPs.

### Quality control

We randomly selected 5% of DNA samples for repeatability testing, and the repeatability of experimental results was >99%.

### Statistical analysis

The differences in demographic characteristics (age, gender etc.) in the present study were tested with SPSS version 21.0 software (SPSS, Chicago, IL, U.S.A.). SPSS software was used to detect whether the four candidate STAT4 SNPs meet Hardy–Weinberg equilibrium. The logistic regression model was used to analyze and calculate odds ratio (OR) and 95% confidence interval (CI), which helped us to predict the association between STAT4 genetic polymorphisms and HCC risk (OR value represents the relative risk; OR = 1: this factor has no effect on HCC risk; OR < 1: this factor can reduce the HCC risk; OR > 1: this factor can increase the HCC risk). Using wildtype alleles as a reference, multiple genetic models are estimated (plink 1.07 online tool software). In the present study, haplotype analysis was conducted by plink1.07 and Haploview software and linkage disequilibrium (LD) was calculated. Finally, we used multifactorial dimensional reduction (MDR) to evaluate the impact of the interaction between candidate SNPs on the HCC risk. All statistical results were adjusted by age and gender and all tests were two-sided tests, and the results of *P*<0.05 were considered statistically significant.

## Results

### Sample characteristics

There is no genetic relationship between all participants in the present study. The average age of the case group (HCC patients) was 55.00 ± 11.56 years, 393 male (78%), 112 female (22%); the average age of the control group was 54.99 ± 10.93 years, and 394 male (78%), 112 female (22%). Table 1 summarized the information for all participants. The statistical results showed that there was no statistical difference between the case and the control group in age (*P*=0.996) and gender (*P*=0.769).

**Table 1:** Characteristics of patients with hepatic carcinoma and healthy individuals

Characteristics	Cases	Control	*P*
	*n*=505	*n*=506	
Age (years)			
Mean ± SD	55.00 ± 11.56	54.99 ± 10.93	0.996
>55	239 (47%)	234 (46%)	
≤55	266 (53%)	272 (54%)	
Gender			
Male	393 (78%)	394 (78%)	0.769
Female	112 (22%)	112 (22%)	
Smoking			
Yes	183 (36%)	231 (46%)	
No	168 (33%)	170 (34%)	
Drinking			
Yes	185 (37%)	132 (26%)	
No	157 (31%)	143 (28%)	

*P*<0.05 indicates statistical significance.

### Genotyping and information of candidate SNPs

We successfully genotyped four candidate genetic loci of STAT4 (rs3821236 A/G, rs11889341 T/C, rs7574865 T/G, rs10174238 G/A). The results showed (Table 2) that all candidate SNPs were consistent with HWE (*P*>5%). The results of HaploReg indicate that the candidate SNPs in the *presentstudy* were regulated by many factors, such as Promoter histone marks; Enhancer histone marks; Motifs changed; NHGRI/EBI GWAS hits; GRASP QTL hits; Selected eQTL hits. The detailed information was shown in Table 2.

**Table 2:** The basic information and HWE about the selected SNPs of *STAT4*

Gene	SNP ID	Function	Chr: Position	Alleles (A/B)	MAF		HWE (*P*-value)	Haploreg 4.1
					Cases	Controls		
STAT4	rs3821236	intronic	2:191038032	A/G	0.400	0.419	0.170	Promoter histone marks; Enhancer histone marks; Motifs changed; NHGRI/EBI GWAS hits; GRASP QTL hits; Selected eQTL hits
STAT4	rs11889341	intronic	2:191079016	T/C	0.280	0.319	0.919	Promoter histone marks; Enhancer histone marks; Motifs changed; NHGRI/EBI GWAS hits
STAT4	rs7574865	intronic	2:191099907	T/G	0.288	0.327	0.685	Enhancer histone marks; Motifs changed; NHGRI/EBI GWAS hits; GRASP QTL hits
STAT4	rs10174238	intronic	2:191108308	G/A	0.260	0.297	0.135	Motifs changed

Abbreviations: HWE, Hardy–Weinberg equilibrium; MAF, minor allele frequency.

*P*>0.05 indicates that the genotypes were in Hardy–Weinberg equilibrium.

### Association between STAT4 candidate SNPs and HCC risk

#### Overall analysis

The results of the association evaluation showed (Table 3) that only STAT4 rs11889341 was associated with HCC susceptibility in the participants and had statistical significance. Specifically, rs11889341 had a significant association with HCC susceptibility reduction among participants under the homozygous (TT vs. CC: OR = 0.6, CI = 0.38–0.96, *P*=0.033), recessive (TT vs. TC-CC: OR = 0.63, CI = 0.40–1.00, *P*=0.028) and log-additive models (OR = 0.83, CI = 0.68–1.00, *P*=0.032). We found no evidence that the remaining three candidate SNPs were associated with HCC risk among participants.

**Table 3:** Analysis of the association between susceptibility of hepatic carcinoma and SNP of *STAT4*

SNP ID	Model	Genotype	Case	Control	Adjusted by age and gender	
					OR (95% CI)	*P*
rs3821236	Allele	A	404	422	0.93 (0.78–1.11)	0.394
		G	606	586	1.00	
	Genotype	AA	73	96	0.78 (0.54–1.13)	0.183
		AG	258	230	1.15 (0.87–1.51)	0.323
		GG	174	178	1.00	
	Dominant	AA-AG	331	326	1.04 (0.8–1.35)	0.772
		GG	174	178	1.00	
	Recessive	AA	73	96	0.72 (0.51–1.00)	0.051
		AG-GG	432	408	1.00	
	Log-additive	-	-	-	0.93 (0.78–1.11)	0.395
rs11889341	Allele	T	281	323	0.83 (0.69–1.00)	0.054
		C	723	689	1.00	
	Genotype	TT	34	52	0.60 (0.38–0.96)	0.033*
		TC	213	219	0.90 (0.69–1.16)	0.406
		CC	255	235	1.00	
	Dominant	TT-TC	247	271	0.84 (0.66–1.08)	0.166
		CC	255	235	1.00	
	Recessive	TT	34	52	0.63 (0.40–1.00)	0.028*
		TC-CC	468	454	1.00	
	Log-additive	-	-	-	0.83 (0.68–1.00)	0.032*
rs7574865	Allele	T	289	328	0.84 (0.69–1.01)	0.063
		G	713	676	1.00	
	Genotype	TT	37	51	0.65 (0.41–1.04)	0.071
		TG	215	226	0.86 (0.66–1.11)	0.253
		GG	249	225	1.00	
	Dominant	TT-TG	252	277	0.82 (0.64–1.05)	0.121
		GG	249	225	1.00	
	Recessive	TT	37	51	0.70 (0.45–1.10)	0.121
		TG-GG	464	451	1.00	
	Log-additive	-	-	-	0.83 (0.68–1.01)	0.059
rs10174238	Allele	G	259	298	0.83 (0.68–1.01)	0.063
		A	739	706	1.00	
	Genotype	GG	28	37	0.68 (0.40–1.15)	0.148
		GA	203	224	0.82 (0.63–1.06)	0.120
		AA	268	241	1.00	
	Dominant	GG-GA	231	261	0.80 (0.62–1.02)	0.072
		AA	268	241	1.00	
	Recessive	GG	28	37	0.75 (0.45–1.24)	0.260
		GA-AA	471	465	1.00	
	Log-additive	-	-	-	0.82 (0.67–1.00)	0.055

*P*<0.05 indicates statistical significance; ‘*’ and bold indicates statistical significance.

#### Age and gender

The results of the age subgroup analysis (Table 4) showed that the candidate SNPs significantly associated with the HCC risk among participants older than 55 years were STAT4 rs11889341, rs7574865 and rs10174238. Similar to the overall analysis result, rs11889341 can significantly reduce the risk of HCC among participants older than 55 years under multiple genetic models (allele: OR = 0.72, CI = 0.54–0.95, *P*=0.020; homozygote: OR = 0.45, CI = 0.22–0.90, *P*=0.024; recessive: OR = 0.50, CI = 0.25–0.99, *P*=0.045; log-additive: OR = 0.71, CI = 0.54–0.95, *P*=0.020). In addition, we also found that rs7574865 can significantly reduce the risk of HCC among participants older than 55 years under allele (OR = 0.70, CI = 0.53–0.93, *P*=0.013), homozygote (OR = 0.40, CI = 0.19–0.83, *P*=0.014), dominant (OR = 0.68, CI = 0.48–1.00, *P*=0.045), recessive (OR = 0.45, CI = 0.22–0.92, *P*=0.028), log-additive genetic models (OR = 0.69, CI = 0.52–0.92, *P*=0.013). rs10174238 also can significantly reduce the risk of HCC among participants older than 55 years under the homozygote (OR = 0.37, CI = 0.15–0.87, *P*=0.023), recessive (OR = 0.39, CI = 0.17–0.91, *P*=0.030), log-additive genetic models (OR = 0.74, CI = 0.54–1.00, *P*=0.045).

**Table 4:** The SNPs of *STAT4* associated with susceptibility of hepatic carcinoma in the subgroup tests (age and gender)

SNP ID	Model	Genotype	Age, years				Gender			
			OR (95% CI)	*P*	OR (95% CI)	*P*	OR (95% CI)	*P*	OR (95% CI)	*P*
			≤55		>55		Female		Male	
rs3821236	Allele	A	0.92 (0.72–1.17)	0.505	0.94 (0.72–1.22)	0.621	0.85 (0.58–1.24)	0.387	0.95 (0.78–1.16)	0.614
		G	1.00		1.00		1.00		1.00	
	Genotype	AA	0.82 (0.5–1.35)	0.438	0.71 (0.41–1.25)	0.241	0.64 (0.30–1.36)	0.246	0.82 (0.54–1.26)	0.375
		AG	1.03 (0.7-1.52)	0.869	1.29 (0.87–1.92)	0.207	1.21 (0.67-2.17)	0.530	1.14 (0.83–1.55)	0.418
		GG	1.00		1.00		1.00		1.00	
	Dominant	AA-AG	0.97 (0.68–1.39)	0.871	1.12 (0.77–1.63)	0.546	1.00 (0.58–1.71)	0.993	1.05 (0.78–1.41)	0.738
		GG	1.00		1.00		1.00		1.00	
	Recessive	AA	0.81 (0.52–1.25)	0.336	0.62 (0.37–1.04)	0.071	0.58 (0.29–1.15)	0.118	0.76 (0.52–1.12)	0.169
		AG-GG	1.00		1.00		1.00		1.00	
	Log-additive	-	0.92 (0.73–1.18)	0.520	0.93 (0.72–1.22)	0.610	0.86 (0.60–1.23)	0.396	0.95 (0.77–1.16)	0.612
rs11889341	Allele	T	0.94 (0.73–1.22)	0.640	0.72 (0.54–0.95)	0.020*	0.88 (0.59–1.32)	0.539	0.81 (0.66–1.01)	0.033*
		C	1.00		1.00		1.00		1.00	
	Genotype	TT	0.80 (0.42–1.51)	0.495	0.45 (0.22–0.90)	0.024*	0.64 (0.24–1.69)	0.366	0.59 (0.35–1.01)	0.013*
		TC	1.03 (0.72–1.46)	0.893	0.78 (0.53–1.14)	0.193	1.00 (0.58–1.73)	0.999	0.87 (0.65–1.16)	0.341
		CC	1.00		1.00		1.00		1.00	
	Dominant	TT-TC	0.99 (0.70–1.39)	0.934	0.71 (0.49–1.02)	0.063	0.93 (0.55–1.57)	0.781	0.81 (0.62–1.08)	0.152
		CC	1.00		1.00		1.00		1.00	
	Recessive	TT	0.79 (0.43–1.46)	0.453	0.50 (0.25–0.99)	0.045*	0.64 (0.25–1.63)	0.347	0.63 (0.38–1.06)	0.042*
		TC-CC	1.00		1.00		1.00		1.00	
	Log-additive	-	0.95 (0.73–1.24)	0.692	0.71 (0.54–0.95)	0.020*	0.88 (0.58–1.32)	0.526	0.81 (0.65–1.01)	0.041*
rs7574865	Allele	T	0.97 (0.75–1.25)	0.805	0.70 (0.53–0.93)	0.013*	0.74 (0.50–1.11)	0.145	0.86 (0.70–1.07)	0.184
		G	1.00		1.00		1.00		1.00	
	Genotype	TT	0.99 (0.54–1.81)	0.962	0.40 (0.19–0.83)	0.014*	0.64 (0.25–1.62)	0.345	0.65 (0.38–1.11)	0.115
		TG	0.96 (0.67–1.37)	0.813	0.77 (0.52–1.12)	0.173	0.63 (0.36–1.11)	0.113	0.93 (0.69–1.25)	0.635
		GG	1.00		1.00		1.00		1.00	
	Dominant	TT-TG	0.96 (0.68–1.36)	0.826	0.69 (0.48–1.00)	0.045*	0.63 (0.37–1.09)	0.097	0.88 (0.66–1.17)	0.373
		GG	1.00		1.00		1.00		1.00	
	Recessive	TT	1.01 (0.56–1.80)	0.981	0.45 (0.22–0.92)	0.028*	0.80 (0.33–1.94)	0.621	0.68 (0.40–1.13)	0.133
		TG-GG	1.00		1.00		1.00		1.00	
	Log-additive	-	0.98 (0.75–1.28)	0.874	0.69 (0.52–0.92)	0.013*	0.73 (0.49–1.10)	0.138	0.86 (0.69–1.07)	0.175
rs10174238	Allele	G	0.90 (0.69–1.17)	0.421	0.76 (0.57–1.02)	0.062	0.83 (0.54–1.27)	0.383	0.83 (0.67–1.04)	0.101
		A	1.00		1.00		1.00		1.00	
	Genotype	GG	1.07 (0.54–2.12)	0.857	0.37 (0.15–0.87)	0.023*	0.75 (0.24–2.37)	0.627	0.66 (0.37–1.19)	0.170
		GA	0.78 (0.55–1.11)	0.171	0.86 (0.59–1.26)	0.442	0.77 (0.44–1.35)	0.365	0.82 (0.62–1.10)	0.194
		AA	1.00		1.00		1.00		1.00	
	Dominant	GG-GA	0.81 (0.58–1.15)	0.239	0.78 (0.54–1.13)	0.184	0.77 (0.45–1.31)	0.339	0.80 (0.60–1.06)	0.122
		AA	1.00		1.00		1.00		1.00	
	Recessive	GG	1.20 (0.62–2.33)	0.592	0.39 (0.17–0.91)	0.030*	0.84 (0.27–2.58)	0.757	0.73 (0.41–1.28)	0.271
		GA-AA	1.00		1.00		1.00		1.00	
	Log-additive	-	0.90 (0.69–1.19)	0.467	0.74 (0.54–1.00)	0.045*	0.82 (0.53–1.27)	0.366	0.82 (0.65–1.03)	0.089

*P*<0.05 indicates statistical significance; ‘-’ indicates Log-additive model; ‘*’ and bold indicates statistical significance.

The results of gender subgroup analysis showed (Table 4) that only STAT4 rs11889341 had a significant association with the reduction in HCC risk among male participants (allele: OR = 0.81, CI = 0.66–1.01, *P*=0.033; homozygote: OR = 0.59, CI = 0.35–1.01, *P*=0.013; recessive: OR = 0.63, CI = 0.38–1.06, *P*=0.042; log-additive: OR = 0.81, CI = 0.65–1.01, *P*=0.041).

#### Smoking and drinking

Participants were also divided according to smoking status. The results showed (Table 5) that STAT4 rs11889341 was only significantly associated with reduction in HCC risk among non-smoking participants under the log-additive model (OR = 0.59, CI = 0.37–0.94, *P*=0.026). However, rs7574865 (allele: OR = 0.51, CI = 0.31–0.82, *P*=0.005; homozygote: OR = 0.26, CI = 0.07–0.92, *P*=0.037; dominant: OR = 0.48, CI = 0.27–0.86, *P*=0.014; log-additive: OR = 0.53, CI = 0.33–0.85, *P*=0.008) and rs10174238 (allele: OR = 0.55, CI = 0.33–0.90, *P*=0.017; heterozygote: OR = 0.50, CI = 0.27–0.95, *P*=0.034; dominant: OR = 0.49, CI = 0.27–0.90, *P*=0.021; log-additive: OR = 0.56, CI = 0.34–0.94, *P*=0.027) were both significantly associated with reducing the HCC risk among non-smoking participants under multiple genetic models.

**Table 5:** The SNPs of *STAT4* associated with susceptibility of hepatic carcinoma in the subgroup tests (smoking and drinking)

SNP ID	Model	Genotype	Smoking				Drinking			
			OR (95% CI)	*P*	OR (95% CI)	*P*	OR (95% CI)	*P*	OR (95% CI)	*P*
			Yes		No		Yes		No	
rs3821236	Allele	A	0.77 (0.52–1.14)	0.186	0.79 (0.52–1.19)	0.253	1.03 (0.66–1.61)	0.903	0.73 (0.49–1.07)	0.109
		G	1.00		1.00		1.00		1.00	
	Genotype	AA	0.63 (0.29–1.41)	0.265	0.46 (0.18–1.17)	0.102	1.06 (0.42–2.71)	0.899	0.42 (0.18–0.96)	0.041
		AG	0.65 (0.35–1.20)	0.165	1.18 (0.63–2.20)	0.610	0.96 (0.47–1.95)	0.910	0.86 (0.47–1.57)	0.613
		GG	1.00		1.00		1.00		1.00	
	Dominant	AA-AG	0.64 (0.36–1.15)	0.134	0.93 (0.51–1.68)	0.806	0.99 (0.50–1.93)	0.965	0.70 (0.40–1.23)	0.218
		GG	1.00		1.00		1.00		1.00	
	Recessive	AA	0.82 (0.40–1.67)	0.579	0.42 (0.17–1.00)	0.051	1.09 (0.47–2.51)	0.841	0.46 (0.21–0.98)	0.055
		AG-GG	1.00		1.00		1.00		1.00	
	Log-additive	-	0.77 (0.52–1.14)	0.192	0.77 (0.52–1.16)	0.218	1.02 (0.64–1.61)	0.936	0.69 (0.47–1.01)	0.057
rs11889341	Allele	T	0.76 (0.50–1.16)	0.201	0.57 (0.36–0.91)	0.018	0.78 (0.47–1.28)	0.329	0.71 (0.47–1.09)	0.116
		C	1.00		1.00		1.00		1.00	
	Genotype	TT	0.70 (0.27–1.83)	0.470	0.27 (0.08–0.98)	0.046	0.61 (0.18–2.05)	0.420	0.52 (0.20–1.39)	0.192
		TC	0.63 (0.35–1.13)	0.121	0.66 (0.36–1.22)	0.188	0.73 (0.37–1.44)	0.365	0.68 (0.38–1.22)	0.197
		CC	1.00		1.00		1.00		1.00	
	Dominant	TT-TC	0.64 (0.37–1.12)	0.118	0.57 (0.32–1.02)	0.059	0.71 (0.37–1.35)	0.295	0.64 (0.37–1.12)	0.118
		CC	1.00		1.00		1.00		1.00	
	Recessive	TT	0.87 (0.35–2.19)	0.772	0.32 (0.09–1.13)	0.077	0.70 (0.22–2.27)	0.556	0.62 (0.24–1.58)	0.315
		TC-CC	1.00		1.00		1.00		1.00	
	Log-additive	-	0.75 (0.49–1.15)	0.190	0.59 (0.37–0.94)	0.026*	0.76 (0.46–1.26)	0.286	0.71 (0.46–1.08)	0.105
rs7574865	Allele	T	0.77 (0.50–1.17)	0.220	0.51 (0.31–0.82)	0.005*	0.87 (0.53–1.41)	0.573	0.58 (0.38–0.90)	0.013*
		G	1.00		1.00		1.00		1.00	
	Genotype	TT	0.67 (0.26–1.73)	0.403	0.26 (0.07–0.92)	0.037*	0.75 (0.24–2.31)	0.617	0.40 (0.14–1.12)	0.081
		TG	0.68 (0.38–1.22)	0.197	0.55 (0.29–1.01)	0.055	0.83 (0.42–1.63)	0.582	0.49 (0.27–0.89)	0.018*
		GG	1.00		1.00		1.00		1.00	
	Dominant	TT-TG	0.68 (0.39–1.18)	0.169	0.48 (0.27–0.86)	0.014*	0.81 (0.43–1.54)	0.524	0.47 (0.27–0.82)	0.008*
		GG	1.00		1.00		1.00		1.00	
	Recessive	TT	0.80 (0.32–1.99)	0.634	0.33 (0.09–1.15)	0.081	0.82 (0.28–2.41)	0.721	0.54 (0.20–1.47)	0.226
		TG-GG	1.00		1.00		1.00		1.00	
	Log-additive	-	0.76 (0.50–1.17)	0.211	0.53 (0.33–0.85)	0.008*	0.85 (0.52–1.39)	0.520	0.57 (0.36–0.88)	0.011*
rs10174238	Allele	G	0.76 (0.50–1.17)	0.210	0.55 (0.33–0.90)	0.017*	0.81 (0.49–1.34)	0.421	0.61 (0.39–0.96)	0.033*
		A	1.00		1.00		1.00		1.00	
	Genotype	GG	0.61 (0.21–1.73)	0.349	0.42 (0.11–1.57)	0.197	0.85 (0.24–3.05)	0.807	0.45 (0.15–1.35)	0.155
		GA	0.71 (0.39–1.26)	0.241	0.50 (0.27–0.95)	0.034*	0.65 (0.33–1.27)	0.206	0.53 (0.29–0.98)	0.041*
		AA	1.00		1.00		1.00		1.00	
	Dominant	GG-GA	0.69 (0.39–1.20)	0.190	0.49 (0.27–0.90)	0.021*	0.67 (0.35–1.29)	0.230	0.52 (0.29–0.91)	0.024*
		AA	1.00		1.00		1.00		1.00	
	Recessive	GG	0.72 (0.26–1.98)	0.522	0.55 (0.15–2.01)	0.369	1.06 (0.31–3.62)	0.928	0.58 (0.20–1.70)	0.318
		GA-AA	1.00		1.00		1.00		1.00	
	Log-additive	-	0.75 (0.48–1.16)	0.193	0.56 (0.34–0.94)	0.027*	0.78 (0.45–1.33)	0.351	0.60 (0.38–0.95)	0.030*

*P*<0.05 indicates statistical significance. ‘-’ indicates Log-additive model; ‘*’ and bold indicates statistical significance.

Participants were divided according to their drinking status. And the results of subgroup showed (Table 5) that STAT4 rs7574865 and rs10174238 had a significant association with the HCC risk among non-drinking participants. Specifically, rs7574865 significantly reduced the HCC risk under the allele (OR = 0.58, CI = 0.38–0.90, *P*=0.013), heterozygote (OR = 0.49, CI = 0.27–0.89, *P*=0.018), dominant (OR = 0.47, CI = 0.27–0.82, *P*=0.008) and log-additive models (OR = 0.57, CI = 0.36–0.88, *P*=0.011). And rs10174238 also significantly reduced the HCC risk under the allele (OR = 0.61, CI = 0.39–0.96, *P*=0.033), heterozygote (OR = 0.53, CI = 0.29–0.98, *P*=0.041), dominant (OR = 0.52, CI = 0.29–0.91, *P*=0.024) and log-additive models (OR = 0.60, CI = 0.38–0.95, *P*=0.030).

### Differences in clinical indicators under different genotypes

We also evaluated the effects of four candidate STAT4 SNPs on the level of clinical indicators under different genotypes. These clinical indicators include carcinoembryonic antigen (CEA), serum ferritin (SF), tumor necrosis factor (TNF), carbohydrate antigen 50 (CA50), carbohydrate antigen 19-9 (CA19-9), carbohydrate antigen 125 (CA125), α-fetoprotein (AFP). The results showed that Table 6) STAT4 rs11889341 (*P*=0.036) and rs7574865 (*P*=0.007) had significant differences in SF levels under different genotypes.

**Table 6:** Clinical characteristics of patients based on the genotypes of selected SNPs

Characteristics	rs3821236				rs11889341			
	AA	AG	GG	*P*	TT	TC	CC	*P*
CEA (ng/ml)	10.64 ± 1.29	14.47 ± 1.65	15.73 ± 1.11	0.197	15.28 ± 4.68	13.9 ± 2.02	14.5 ± 0.72	0.917
SF (ng/ml)	94.07 ± 7.47	90.89 ± 1.93	89.63 ± 1.96	0.675	105.02 ± 13.79	91.26 ± 2.55	88.77 ± 1.46	0.036*
TNF (mol/ml)	0.9 ± 0.01	0.9 ± 0.01	0.89 ± 0.01	0.786	0.89 ± 0.02	0.89 ± 0.01	0.9 ± 0.01	0.668
CA50 (U/ml)	7.08 ± 1.37	10.55 ± 1.9	8.6 ± 1.38	0.507	9.41 ± 1.83	8.62 ± 1.58	9.7 ± 1.65	0.901
CA125 (U/ml)	77.74 ± 24.58	104.69 ± 19.06	85.02 ± 19.17	0.654	45.29 ± 30.97	98.93 ± 20.6	88.5 ± 15.2	0.638
CA199 (U/ml)	32.31 ± 5.19	60.78 ± 10.6	58.1 ± 10.13	0.290	48.66 ± 12.24	50.27 ± 10.83	60.04 ± 8.95	0.737
AFP (ng/ml)	45.65 ± 21.08	34.68 ± 7.77	62.62 ± 13.69	0.200	82.65 ± 44.18	35.11 ± 9.02	49.8 ± 9.67	0.201

Characteristics	rs7574865				rs10174238			
	TT	TG	GG	*p*	GG	GA	AA	*P*
CEA (ng/ml)	13.1 ± 4.05	12.55 ± 1.08	14.56 ± 0.73	0.358	15.82 ± 1.48	12.52 ± 0.99	11.26 ± 3.48	0.179
SF (ng/ml)	105.02 ± 13.79	87.58 ± 1.7	89.62 ± 1.22	0.007*	91.18 ± 1.88	90.63 ± 2.97	89.63 ± 3.25	0.975
TNF (mol/ml)	0.89 ± 0.02	0.89 ± 0.01	0.9 ± 0.01	0.871	0.9 ± 0.01	0.9 ± 0.01	0.87 ± 0.02	0.543
CA50 (U/ml)	9.41 ± 1.83	8.18 ± 1.46	9.8 ± 1.76	0.788	8.48 ± 1.07	10.6 ± 2.29	9.91 ± 3.09	0.646
CA125 (U/ml)	44.41 ± 22.55	103.97 ± 21.1	88.41 ± 15.7	0.469	86.77 ± 14.08	108.1 ± 23.55	58.76 ± 31.2	0.576
CA199 (U/ml)	42.94 ± 11.41	50.38 ± 10.65	61.25 ± 9.39	0.611	58.95 ± 8.55	53.41 ± 11	34.47 ± 12.53	0.663
AFP (ng/ml)	70.67 ± 38.49	40.26 ± 9.84	48.15 ± 9.68	0.507	45.92 ± 9.2	50.37 ± 12.15	16.83 ± 10.83	0.567

AFP, alpha-fetoprotein; CA19-9, carbohydrate antigen 19-9; CA50, carbohydrate antigen 50; CA125, carbohydrate antigen 125; CEA, carcinoembryonic antigen; SF, serum ferritin; TNF, tumor necrosis factor; ‘*’ and bold indicates statistical significance.

### Analysis of MDR

**Figure 1:**
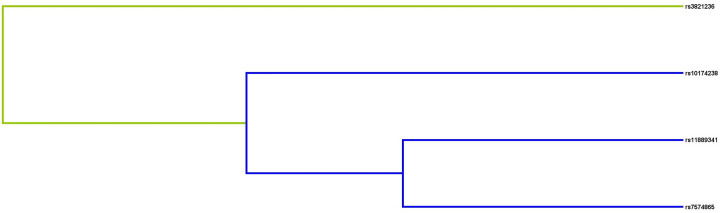
Dendrogram analysis of SNP–SNP interaction The colors in the tree diagram represent synergy (yellow) or redundancy (blue).

**Figure 2:**
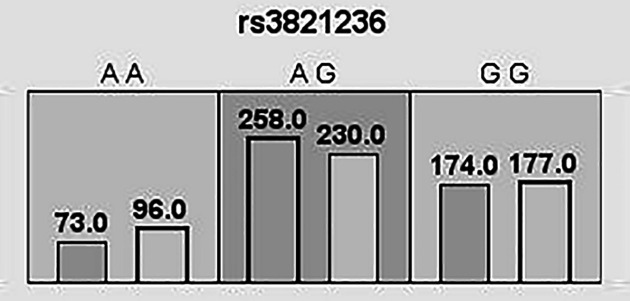
MDR analysis of STAT4 rs3821236 interaction In each box, the left bar represents cases and the right bar represents controls. The light gray lattice indicates the low risk of HCC and dark gray lattice indicates the high risk of HCC, the empty lattice means no data.

**Figure 3:**
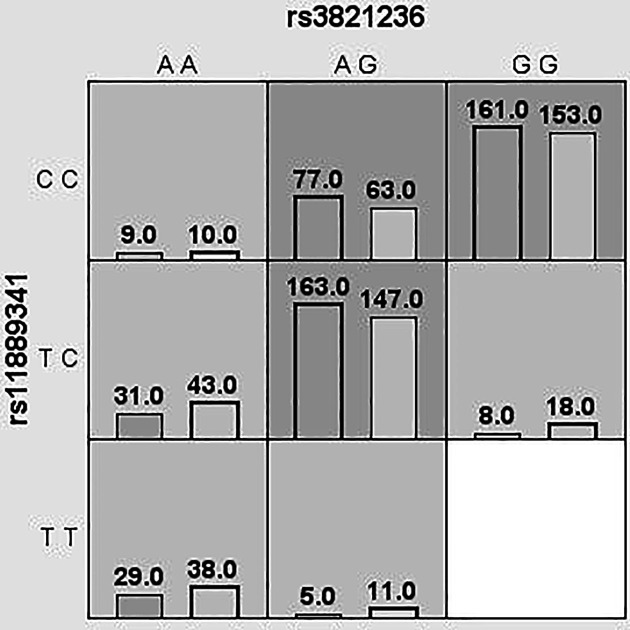
MDR analysis of STAT4 rs3821236–rs11889341 interaction In each box, the left bar represents cases and the right bar represents controls. The light gray lattice indicates the low risk of HCC and dark gray lattice indicates the high risk of HCC, the empty lattice means no data.

**Figure 4:**
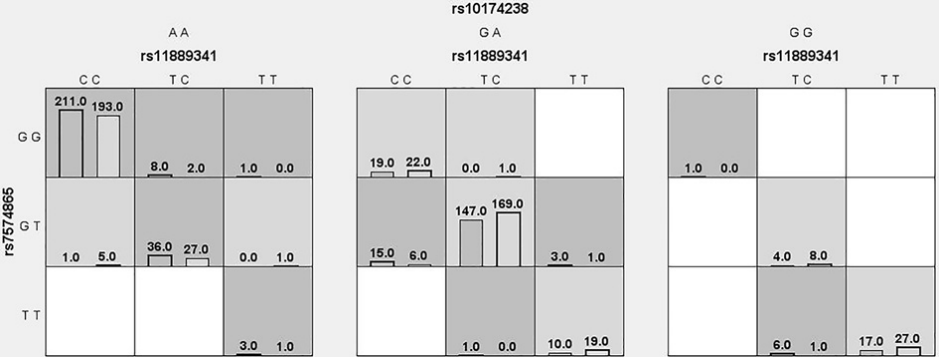
MDR analysis of STAT4 rs11889341–rs7574865–rs10174238 interaction In each box, the left bar represents cases and the right bar represents controls. The light gray lattice indicates the low risk of HCC and dark gray lattice indicates the high risk of HCC, the empty lattice means no data.

**Figure 5:**
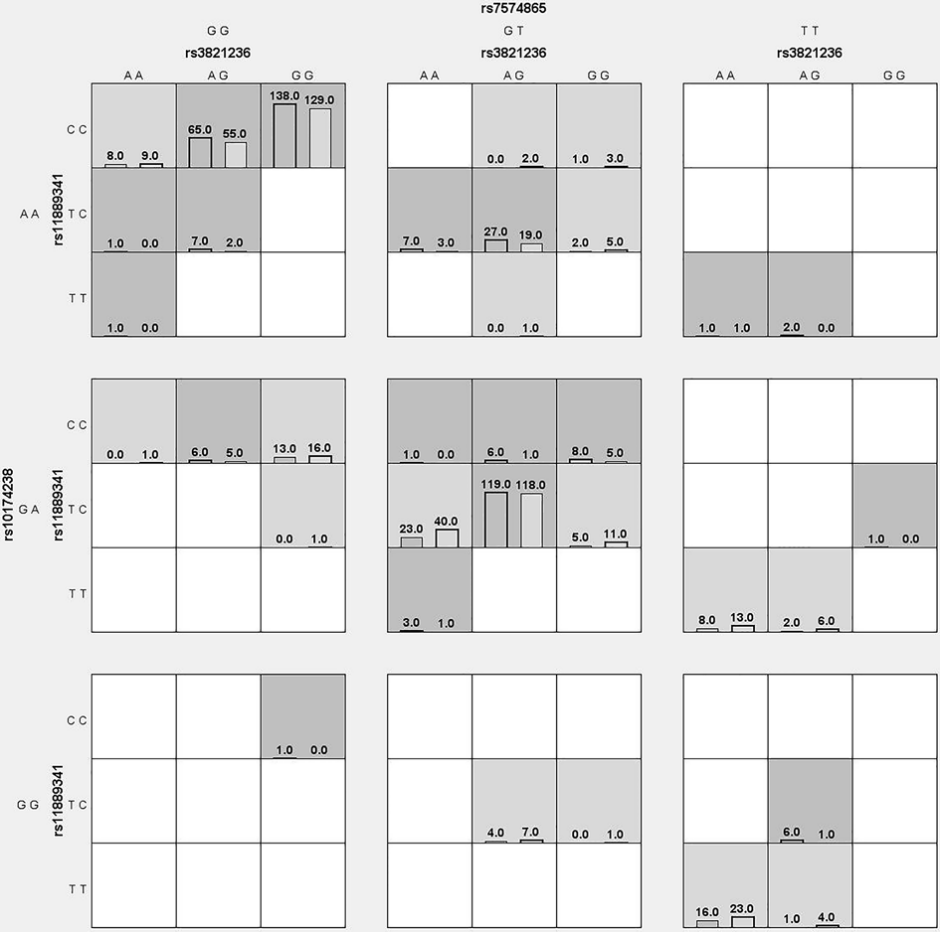
MDR analysis of STAT4 rs3821236–rs11889341–rs7574865–rs10174238 interaction In each box, the left bar represents cases and the right bar represents controls. The light gray lattice indicates the low risk of HCC and dark gray lattice indicates the high risk of HCC, the empty lattice means no data.

We used MDR to analyze and evaluate the interaction of candidate SNPs in HCC risk among participants. Figure 1 was a dendrogram analysis of SNP–SNP interaction. The blue line in Figure 1 indicated that candidate SNPs have redundant effects in regulating HCC susceptibility, and the yellow line indicated synergy effects. The results were shown in Table 7. The best two-site model for predicting the HCC risk is: rs3821236, rs11889341 (testing accuracy = 0.512, cross-validation consistency = 8/10, *P*=0.0004); the three-site model is: rs11889341, rs7574865, rs10174238 (testing accuracy = 0.523, cross-validation consistency = 8/10, *P=*0.0003); the four-site model is: rs3821236, rs11889341, rs7574865, rs10174238 (testing accuracy = 0.527, cross-validation consistency = 10/10, *P*<0.0001). The interaction of ‘SNP–SNP’ in different loci model combinations can be seen in Figures 2-5. Among them, light gray lattice represented a low risk of HCC, dark gray lattice represented a high risk of HCC and no color filled lattice represented no data. The results revealed that the effect of four candidate SNPs on HCC risk may be interdependent.

**Table 7:** SNP–SNP interaction models analyzed by the MDR method

Model	Training Bal. Acc	Testing Bal. Acc	OR (95% CI)	*P*-value	CVC
rs3821236	0.533	0.482	1.25 (0.98–1.60)	0.0779	5/10
rs3821236, rs11889341	0.546	0.512	1.74 (1.28–2.36)	**0.0004**	8/10
rs11889341, rs7574865, rs10174238	0.559	0.523	1.59 (1.24–2.04)	**0.0003**	8/10
rs3821236, rs11889341, rs7574865, rs10174238	0.570	0.527	2.18 (1.61–2.94)	**<0.0001**	10/10

Abbreviations: Bal. Acc., balanced accuracy; CVC, cross-validation consistency. *P*-values were calculated using χ^2^ tests; *P*<0.05 indicates statistical significance; bold indicates statistical significance.

### Haplotype analysis

**Figure 6:**
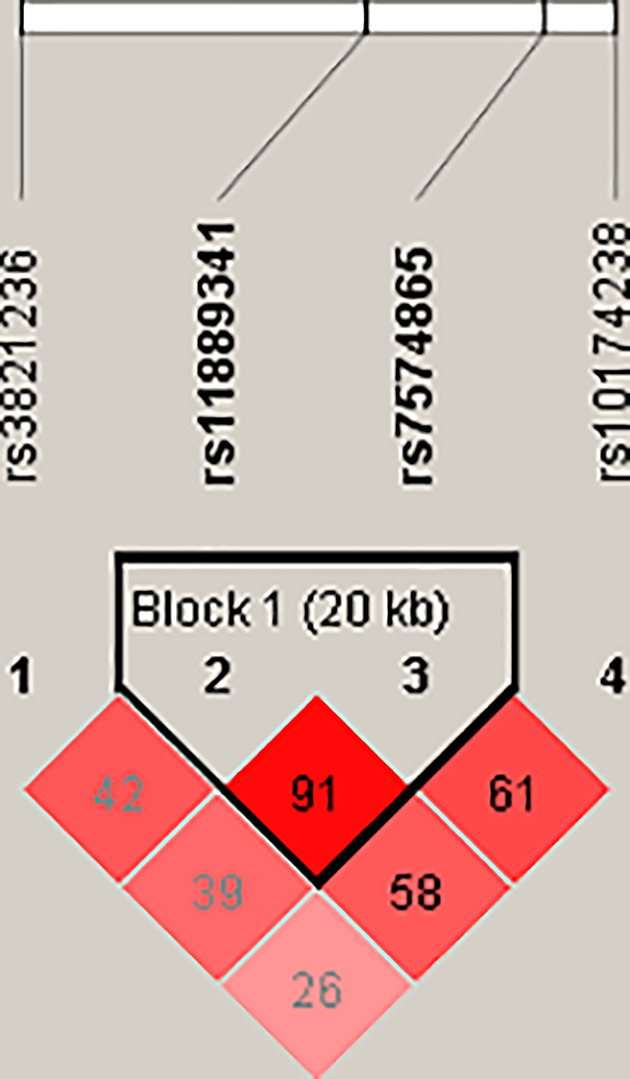
LD plots containing four polymorphisms from *STAT4* The lighter the color, the lower the degree of linkage. The numbers inside the diamonds indicate the D′ for pairwise analyses.

The results of LD and haplotype analysis of STAT4 polymorphism showed (Figure 6): there is an LD block (D′ = 0.976, R^2^ = 0.919) composed of two SNPs (rs11889341 and rs7574865). Table 8 summarized the frequency of haplotypes formed by STAT4 genetic polymorphisms in the case and control groups. In haplotype analysis, the covariates (age and gender) were adjusted. Logistic regression results showed that the haplotype ‘TT’ (rs11889341|rs7574865) can reduce the susceptibility of HCC among participants (crude analysis: OR = 0.81, CI = 0.66–0.99, *P*=0.037; adjusted by age and gender: OR = 0.81, CI = 0.66–0.99, *P*=0.130).

**Table 8:** Haplotype frequencies and the association with the risk of HCC

Chromosome	Gene	SNP	Haplotype	Frequency		Crude analysis		Adjusted by age and gender	
				Cases	Controls	OR (95% CI)	*P*	OR (95% CI)	*P*
Chr2	STAT4	rs11889341|rs7574865	CG	0.697	0.668	1.00	-	1.00	-
		rs11889341|rs7574865	TT	0.266	0.314	0.81 (0.66–0.99)	0.036*	0.81 (0.66–0.99)	0.037*
		rs11889341|rs7574865	CT	0.023	0.013	1.71 (0.85–3.42)	0.130	1.71 (0.85–3.44)	0.130

*P*<0.05 indicates statistical significance; ‘*’ and bold indicates statistical significance.

## Discussion

Studies have confirmed that individual genetic variation affects the occurrence and development of HCC [16]. STAT4 is an important transcriptional activator of JAK/STAT signaling pathway, and plays an important role in the body’s immune response, antiviral infection, and promotion of tumor cell invasion and metastasis. STAT4 may be a breakthrough in the prevention and treatment of HCC [17]. Therefore, in-depth study of STAT4 gene polymorphisms, function and expression will help to understand the mechanism of occurrence and development of HCC, and also provide a theoretical basis for the treatment and prevention of HCC.

Our study conducted a ‘case–control’ study among the Chinese Han population. In general, only STAT4 rs11889341 has a significant association with the HCC risk in the present study subjects under multiple genetic models (homozygote: *P*=0.033; recessive: *P*=0.028; log-additive: *P*=0.032), and it has shown the ability to reduce the HCC risk. The remaining three candidate SNPs were not associated with the HCC risk in the study subjects. As far as we know, we are the first to report that STAT4 rs11889341 is associated with disease risk.

In previous studies, the association between STAT4 rs7574865 and HCC risk has been reported many times [18–20]. But our results are not exactly the same as previous studies. In the overall analysis, rs7574865 was not associated with HCC susceptibility among participants. But in the subgroup analysis, it showed the ability to reduce the HCC susceptibility among specific population (participants older than 55 years, non-smokers or non-drinking alcohol). The results of our study are similar to previous studies: Chen et al. have reported that rs7574865 (OR = 0.79) is associated with a reduction in the risk of HCC [14]. However, a meta-analysis of eight studies revealed that the rs7574865 polymorphism may be used as one of the risk factors for HCC [15], which is contrary to the results of our study. We speculate that the reasons for the above differences may be caused by differences in sample size or genetic background. In any case, our study once again verified that there is a certain association between STAT4 rs7574865 and HCC susceptibility.

In previous studies, factors related to the risk of HCC included aging [21], smoking [22–24] and alcohol consumption [22,25,26]. And some studies have reported that there are gender differences in the incidence of HCC [27,28]. Therefore, we also divided the study subjects according to the current epidemiological characteristics of HCC or the difference in risk of incidence (age, gender, smoking/drinking status). Then, we analyzed the association between candidate SNPs and HCC risk in subgroups, with a view to provide a valuable reference for the prediction or evaluation of HCC risk in specific populations. Our results showed that STAT4 rs11889341 is significantly associated with reduction in HCC risk among participants who were older than 55 years, male or non-smoker. Both rs7574865 and rs10174238 were significantly associated with reduction in HCC risk among participants who were older than 55 years, did not smoke or drink alcohol. On the one hand, previous studies have confirmed that the incidence of HCC among non-smokers/drinkers is lower than that among smokers/drinkers [22,29]. Combined with the results of our study, we speculate that STAT4 rs11889341, rs7574865 or rs10174238 may inhibit HCC among non-smoking or non-drinking participants. But their specific mechanism in HCC needs further experimental to verify. On the other hand, rs11889341 was significantly associated with reduction in HCC risk among potentially HCC-susceptible populations (older than 55 years old/male participants) in the present study. It has been confirmed that older population [21] or males [28] are more likely to develop HCC. Based on this, we speculate that STAT4 rs11889341 may be a protective factor for HCC in the Chinese Han population, and this protective effect may not be affected by non-biological risk factors. However, a large sample size and further verification tests are necessary to ensure the accuracy of our results. Nevertheless, our study is the first report on the potential association between STAT4 rs11889341, rs7574865 or rs10174238 and HCC risk among Chinese Han population. Our study has provided new evidence for predicting the targeted therapy of HCC and the exact molecular mechanism of STAT4 in the occurrence and development of HCC, and also provided new ideas for the role of STAT4 in the prevention and treatment of HCC.

STAT4 is an important transcriptional activator. After activation, it crosses the nuclear membrane into the nucleus in the form of a homodimer and initiates the transcription and expression of downstream target genes [30]. Studies have shown that STAT4 is involved in the occurrence and development of HCC and other tumors, and the abnormal expression of STAT4 is closely related to tumor metastasis and prognosis [10,11]. Studies have also shown that chemotherapy-induced STAT4 deficiency can help lymphoma patients transplanted with peripheral blood stem cells to produce IFN-γ, thereby inhibiting the growth of tumor cells [31]. Based on the above studies, the expression level of STAT4 can directly affect the occurrence and development of tumors. Combined with the results of our study, rs11889341, rs7574865 or rs10174238 and the reduction in HCC risk among the participants may be caused by these genetic variants affecting the expression level of STAT4. We may be able to start with the effect of these variants on the expression level of STAT4 in the occurrence and development of HCC, and further explore the molecular mechanism of STAT4 in the occurrence and development of HCC. We believe it will be very interesting.

SF as a potential cancer marker has been confirmed by many studies [32,33]. Although there were studies found that SF levels may not play a role in the identification and diagnosis of HCC [34,35], other studies have also shown that it may be a marker for monitoring chemotherapy response in patients with HCC [33]. Facciorusso et al. also found that the prognosis of HCC patients with higher SF levels was poor [36]. In this study, we found that the SF levels under different genotypes of STAT4 rs11889341, rs7574865 had significant differences. Combined with previous studies, our results suggest that genetic variants in STAT4 can affect SF levels, thereby affecting the risk of HCC. Our study may provide a new reference for clinical monitoring of HCC.

Our study provides data supplements for the study of association between STAT4 gene polymorphisms and HCC risk in Chinese Han population. In particular, rs11889341 showed an outstanding and significant association with the risk of HCC. However, we must face the fact that the present study has certain limitations. In order to increase the reliability and repeatability of the results, a large sample size is indeed necessary. At present, only a small part of STAT4 genetic sites associated with the risk of HCC have been discovered, and there are more genetic susceptibility sites/regions that need new research to discover. With the continuous exploration of the susceptible sites of HCC in the future, it is of great help for individualized treatment and diagnosis of HCC in clinic.

## Conclusion

In summary, we found that STAT4 rs11889341, rs7574865 or rs10174238 had a potential association with the reduction in HCC risk in Chinese Han population. Especially rs11889341, whether in the overall analysis or subgroup analysis, it has significant association with reducing the risk of HCC.

## Data Availability

The datasets used and analyzed in the current study are available from the corresponding author on reasonable request.

## Abbreviations

CI: confidence interval
HBV: hepatitis B virus
HCC: hepatocellular carcinoma
LD: linkage disequilibrium
MDR: multifactorial dimension reduction
OR: odds ratio
SF: serum ferritin
SNP: single nucleotide polymorphism
STAT4: signal transducer and activator of transcription 4
